# Otolaryngology Away Subinternships: An Analysis of the Application Process and Survey of Current Applicants' Perspectives

**DOI:** 10.1002/oto2.49

**Published:** 2023-03-28

**Authors:** Thomas Cyberski, Swetha Tatineni, Andrea Shogan, Fuad M. Baroody

**Affiliations:** ^1^ Pritzker School of Medicine and Section of Otolaryngology–Head and Neck Surgery The University of Chicago Chicago Illinois USA; ^2^ Section of Otolaryngology–Head and Neck Surgery, Department of Surgery The University of Chicago Medicine Chicago Illinois USA

**Keywords:** match, otolaryngology, residency, subinternship

## Abstract

**Objective:**

To assess the availability and uniformity of application information for away subinternships and survey 4th‐year medical students on their experiences obtaining away subinternships in otolaryngology‐head and neck surgery (OHNS) during the 2022 to 2023 application cycle.

**Study Design:**

Cross‐sectional study.

**Setting:**

Online survey.

**Methods:**

The Association of American Medical College's Visiting Student Learning Opportunities (VSLO) program was queried for information on OHNS away subinternship applications. A survey assessing 4th‐year medical students' perceptions of the away subinternship application process was distributed via OHNS residency program directors and Otomatch.

**Results:**

Of 129 OHNS residency programs, 103 (80%) offered away subinternship opportunities on VSLO. Variability in application release dates (January 18 to June 3, 2022), offer release dates (January 27 to August 7, 2022), and estimated cost ($22‐$5500) were found. The most common application requirements were a transcript (98.1%) and a CV/resume (90.3%). There were 64 survey respondents, for a 13% response rate. The most common concerns include applying to too few programs (80%) and not knowing offer release dates (77%). The most common stressors include choosing a number of programs to which to apply (48%) and cost (35%). The majority (76%) reported difficulty finding updated information on program websites. Among the proposed changes, the greatest support was found for having all applications on VSLO (88%), uniform application release date (84%), and uniform application requirements (82%).

**Conclusion:**

The OHNS away subinternship application process is a significant source of anxiety for medical students due to the tremendous variability in application and acceptance procedures. Having all applications on VSLO, uniform application requirements, and uniform application opening and offer release dates would better facilitate this process.

Otolaryngology‐head and neck surgery (OHNS) is among the most competitive specialties. In the 2022 Match cycle, the National Resident Matching Program reported that 574 medical students applied for 361 residency positions, yielding a 63% match rate.^1^ Given the recent transitioning of the US Medical Licensing Examination (USMLE) step 1 exam to pass/fail and the implementation of the preference signaling system, OHNS residency programs will likely prioritize different applicant characteristics during future match cycles.^2^, ^3^ In particular, away subinternships (also referred to as audition electives, acting internships, or externships) will likely play a more important role.^4^, ^5^

Away subinternships are a staple of the 4th‐year medical school curriculum, especially for students applying to highly competitive specialties. These 2‐ to 4‐week rotations allow for educational experiences outside students' home institutions and the chance for both programs and applicants to assess “fit.” Programs consistently report personal knowledge of applicants as among the most important criteria used when ranking applicants, and in cases of exceptional “fit,” match rates as high as nearly 50% are reported.^4^, ^6^ With the creation of the Association of American Medical College's (AAMC's) Visiting Student Learning Opportunities (VSLO) program, applying for these opportunities has become more streamlined in some respects. However, rotation calendars, application requirements, and other aspects of this process remain highly variable, leading to significant stress among students.

Recent studies have characterized away subinternships in several fields, including orthopedic surgery, emergency medicine, plastic surgery, and OHNS.^4^, ^7^, ^8^, ^9^, ^10^, ^11^ These studies, however, mainly investigate the utility of away subinternships in the residency application process, with few studies focusing on the process of securing an away subinternship itself and even fewer considering 4th‐year medical students' perspectives.^8^, ^10^, ^11^ The objectives of this study are to (1) assess the availability and uniformity of OHNS away subinternship application‐related information for all programs offering such opportunities and (2) survey 4th‐year medical students about their application experiences and potential areas of improvement.

## Methods

### Away Subinternship Application Analysis

Using the Accreditation Council for Graduate Medical Education (ACGME) list of 129 accredited US OHNS residency programs, we queried the AAMC's VSLO website on June 9, 2022 for away subinternships offered by each program. We performed the search on VSLO under the “Find Electives” tab with a filter added for Specialty: Otolaryngology. In the search bar, we searched for electives offered by each program either by the program's name as it is listed on the ACGME list or its location (“city, state”).

For programs with away subinternships on VSLO, data were collected regarding application timelines, offer release dates, processing or tuition fees, total estimated cost (including accommodations, transportation, and food), and points of contact. Each application was also queried for program‐specific requirements which included: CV/resume, transcript, USMLE scores, letter of recommendation, personal statement, AAMC immunization form, immunization titers, current tuberculosis (TB) test results, mask fit results, photograph, background check, basic life support/advanced cardiac life support certification, drug screen results, malpractice insurance, and personal health insurance.

Electives offered by programs not utilizing VSLO were found using the Google search “[program name] away subinternship application.” Information about these electives was gathered, including whether the program has a specific webpage and application system.

### Survey Development

Survey questions assessing students' perceptions of the away subinternship application process were developed after a literature review and discussions with current otolaryngology applicants at the Pritzker School of Medicine, University of Chicago. Our survey assessed student demographic characteristics (eg, gender, race/ethnicity, age, marital status, number of children, region of home medical school, preferred subinternship region, and number of planned subinternships), application process characteristics (eg, factors considered while applying, ability to find relevant information, and perceived stress/concerns), and areas of improvement. We piloted the survey with 5 senior medical students, and their feedback on clarity, appropriateness, and organization was incorporated. The final, anonymous electronic survey consisted of 46 items with quantitative and qualitative questions, including the Likert scale, multiple choice, and free‐response questions. The institutional review board at the University of Chicago (IRB22‐0736) approved the study (see Supplemental Appendix, available online).

### Survey Distribution

In May 2022, we distributed the survey to a national electronic listserv for otolaryngology residency program directors who were asked to email the survey link to their respective senior medical students. We also posted the survey on Otomatch, an online forum for otolaryngology applicants. Reminders were sent to both platforms after 2 weeks. The survey was open for 4 weeks between May and June 2022. All US senior medical students in allopathic or osteopathic medical schools applying to otolaryngology residency programs during the 2022 to 2023 application cycle were eligible. Participation was voluntary, and data was collected using REDCap, a secure application for managing online surveys (v11.1.7, 2022 Vanderbilt University).

### Data Analysis

Standard descriptive statistics were used for analysis. Only completed surveys from 4th‐year otolaryngology‐bound US medical students were considered. *T* tests and 1‐way analysis of variance examined associations between application process stressors with covariates such as race, gender, and access to home ENT program after converting ordinal data to numbers (ie, not at all stressed corresponds to 1 and extremely stressed corresponds to 5). Two authors (T.C. and S.T.) independently generated common themes from survey free‐response questions. All authors finalized themes, and theme frequencies were calculated using keywords related to a theme. All analyses were performed in STATA 16.0 (StataCorp, 2019), with a *p* < .05 determined as significant.

## Results

### Away Subinternship Application Analysis

**Figure 1:**
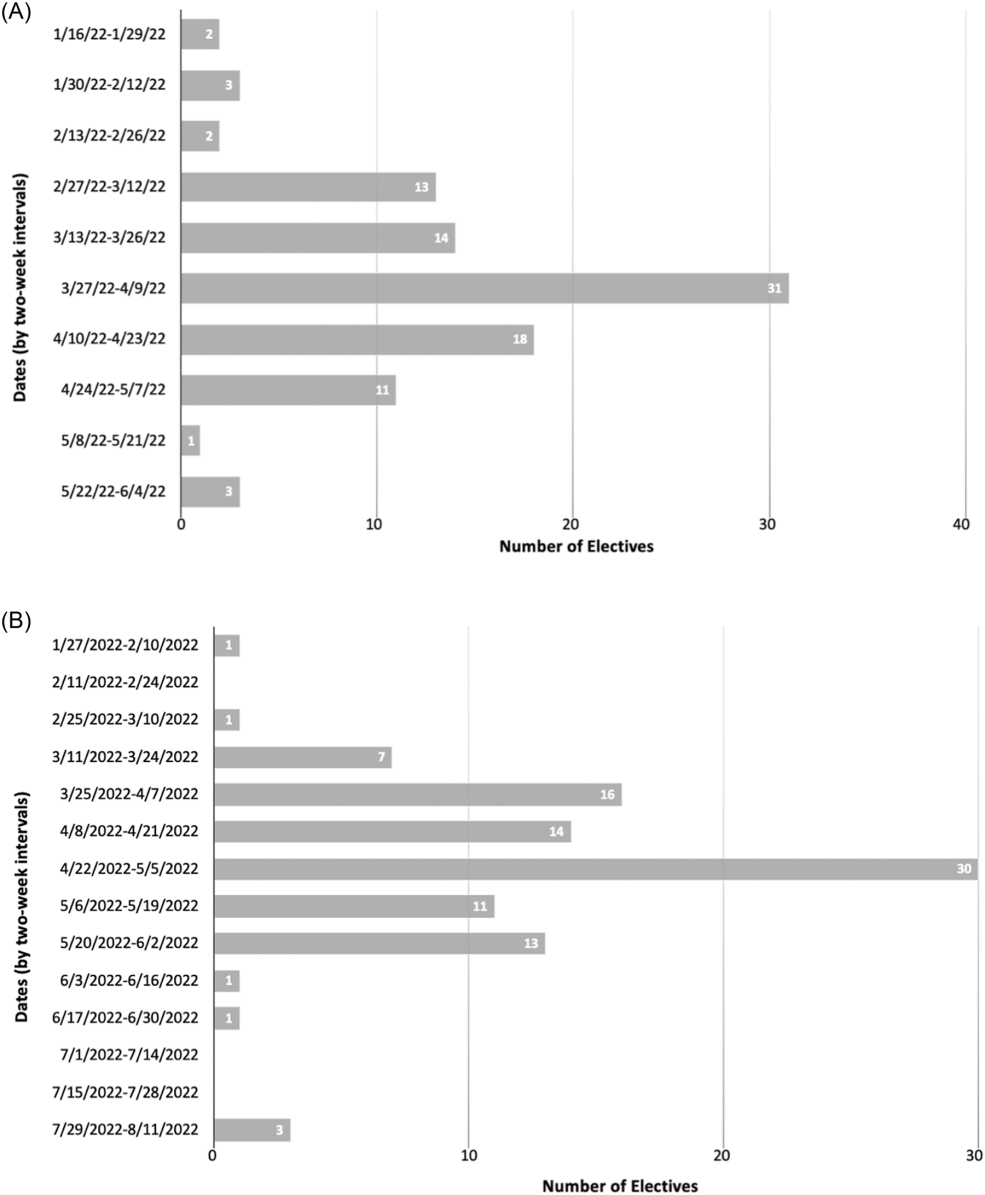
Graphical representations of the dates applications for away subinternships open on VSLO (A) and dates that offer of acceptance for away subinternships begin to be extended (B). One school did not have any dates available and 4 programs' dates were outdated. VSLO, Visiting Student Learning Opportunities.

Of 129 accredited US OHNS residency programs, 103 (80%) offered away subinternships on VSLO. Of these electives, the dates that applications opened ranged from January 18, 2022 to June 3, 2022, with the most frequent date being April 1, 2022 (19 programs, 18.4%). Offer release dates ranged from January 27, 2022 to August 7, 2022, with the most frequent date being May 2, 2022 (15 programs, 14.6%) (Figure 1). Sixty‐two (60.2%) programs required a processing or tuition fee, ranging from $25 to $300. Forty‐one (39.8%) programs provided an estimated total cost of attendance (including tuition/fees, housing, transportation, etc) (Table 1). Total cost averaged $1537.16, not including 7 programs with an estimated cost of less than $100. Eighty‐four (81.6%) programs named specific contact persons.

**Table 1:** VSLO Data

	Total (%)	Range
Number of US OHNS programs on VSLO	103 (80.0)	
Most frequent date applications open	April 1, 2022 (18.4)	January 18 to June 3, 2022
Most frequent date offers released	May 2, 2022 (14.6)	January 27 to August 7, 2022
Programs with processing or tuition fees	62 (60.2)	$0‐$300
Programs with an estimated cost	41 (39.8)	$22‐$5500
Average estimated cost^a^	$1537.16	
Programs with a specific point of contact	84 (81.6)	
Most frequent application requirements^b^		
Transcript	101 (98.1)	
CV/resume	93 (90.3)	
Photograph	93 (90.3)	
AAMC immunization form	91 (88.4)	
USMLE scores	85 (82.5)	
Background check	49 (47.6)	
Malpractice insurance	43 (41.8)	
Personal statement	32 (31.1)	
Health insurance	32 (31.1)	
ACLS/BLS certification	30 (29.1)	
Mask fit	28 (27.2)	
Immunization titers	22 (21.4)	
Drug screen	20 (19.4)	
Letter of reccomendation	17 (16.5)	
TB test	10 (9.7)	

Abbreviations: AAMC, Association of American Medical Colleges; ACLS/BLS, advanced cardiac life support/basic life support; OHNS, otolaryngology‐head and neck surgery; TB, tuberculosis; USMLE, US Medical Licensing Examination; VSLO, Visiting Student Learning Opportunities.

^a^ Average estimated cost among the schools with an estimated cost of ≥$100.

^b^ As reported on the individual VSLO elective pages.

Application requirements for each away subinternship on VSLO were also assessed. The most frequently required application materials were transcript (101, 98.1%), CV/resume (93, 90.3%), photograph (93, 90.3%), AAMC immunization form (91, 88.4%), and USMLE scores (85, 82.5%). The least frequently required application materials were current TB test (10, 9.7%), letter of recommendation (17, 16.5%), drug screen (20, 19.4%), immunization titers (22, 21.4%), and mask fit (28, 27.2%) (Table 1).

Twenty‐six (20.2%) OHNS residency programs were not found to offer away subinternships on VSLO. Using the search criteria outlined above, 23 (88.5%) of these programs had a webpage for away subinternship opportunities, and 14 (53.8%) had a specific application. Of the programs without electives on VSLO, 13 (50%) are affiliated with either military or osteopathic schools. Notably, several well‐known OHNS residency programs were found to utilize their own application systems for away subinternships, including Johns Hopkins University, Stanford University, University of Iowa, University of Pennsylvania, Cleveland Clinic Foundation, and NYU Grossman School of Medicine.

### Applicant Survey

Electronic Residency Application Service (ERAS) preliminary data suggested an estimated total of 490 US MD or DO OHNS applicants for the 2023 Match cycle. A total of 64 surveys were completed and included in our analysis, yielding an estimated 13% response rate. While age and gender were representatives of national medical school enrollment data, our study had more Asian students (44%), fewer black students (3.1%), and fewer students from the AAMC Western region (14.1%).^12^ Students applied to an average of 5.2 (range 1‐12) institutions. Table 2 summarizes respondent characteristics.

**Table 2:** Characteristics of 64 US Medical Student Respondents

	N (%) or mean (range)
Gender	
Female	33 (51.5)
Male	30 (46.8)
Nonbinary	1 (1.6)
Race and ethnicity	
Hispanic	5 (7.8)
Non‐Hispanic	59 (92.2)
Asian	28 (43.8)
White	25 (39.1)
Mixed	5 (7.8)
Black/African American	2 (3.1)
American Indian/Alaskan Native	1 (1.6)
Native Hawaiian/Pacific Islander	0
Other	2 (3.1)
Missing	1 (1.6)
Age	26 (23‐33)
Marital status	
Single, never married	53 (82.8)
Married or domestic partnership	9 (14.1)
Widowed	1 (1.6)
Missing	1 (1.6)
Number of children	
0	58 (90.6)
1‐2	5 (7.8)
3+	1 (1.6)
Region of home medical school	
Southern	20 (31.3)
Central	18 (28.1)
Northeastern	17 (26.6)
Western	9 (14.1)
Have a home OHNS program	58 (90.6)
Number of desired away rotations per applicant	2 (1‐4)
Number of applications per applicant	5.2 (1‐12)
First choice region to do away with rotation	
Northeastern	24 (37.5)
Western	17 (26.6)
Southern	12 (18.8)
Central	11 (17.2)

Central—IL, IN, IA, KS, MI, MN, MO, NE, ND, OH, SD, WI; Northeastern—CT, DE, ME, MD, MA, NH, NJ, NY, PA, RI, VT, DC; Southern—AL, AR, FL, GA, KY, LA, MS, NC, OK, PR, SC, TN, TX, VA, WV; Western—AK, AZ, CA, CO, HI, ID, MT, NV, NM, OR, UT, WA, WY.

Abbreviation: OHNS, otolaryngology‐head and neck surgery.

Table 3 lists the top 5 reasons for selecting a particular away subinternship and the top 5 concerns while applying for away subinternships. Totals do not add up to 100% as respondents selected and ranked their top 5 reasons and concerns among a list of 15 and 10, respectively (see Supplemental Appendix, available online). Geographic location (97%) was the most commonly selected reason for choosing an away subinternship. Other factors considered were program reputation (69%), available rotation dates (67%), family/friends in the area (58%), and size of the program (44%). Notably, geographic location (44%) was also the most frequently ranked number 1 reason for choosing an away subinternship, followed by the size of the program (8%) and prior exposure to program (8%).

**Table 3:** Top 5 Reasons for Selecting a Particular Away Subinternship and Top 5 Concerns While Applying for Away Subinternships

	N (%)
Reasons for electing a subinternship	
Geographic location(s)	62 (97)
Prestige of program(s)	44 (69)
Available rotation dates	43 (67)
Family/friends in the area(s)	37 (58)
Size of program(s)	28 (44)
Concerns while applying	
Applying to too few programs	51 (80)
Not knowing when offers would be released	49 (77)
Not receiving enough offers	47 (73)
Applying to too many programs	39 (61)
Overall cost	28 (44)

Applying to too few programs (80%) was the most commonly selected concern while applying for away subinternships. Other concerns included not knowing offer release dates (77%), not receiving enough offers (73%), applying to too many programs (61%), and overall cost (44%). Notably, not knowing offer release dates (22%) was the most frequently ranked number 1 concern, followed by applying to too few programs (19%) and applying to too many programs (16%).

**Figure 2:**
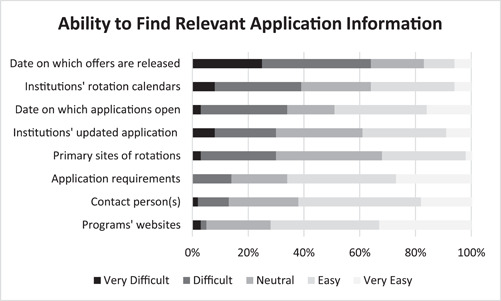
Graphical representation of perceived abilities to find relevant away subinternship application information as reported by survey respondents (n = 64).

Figure 2 shows applicants' perceived abilities to find application information. The majority of applicants (64%) indicated difficulty finding offer release dates, followed by institutions' rotation calendars (39%) and application release dates (34%).

**Figure 3:**
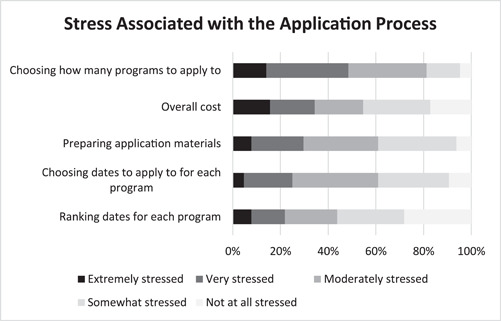
Graphical representation of perceived levels of stress associated with various components of the away subinternship application process as reported by survey respondents (n = 64).

Figure 3 shows applicants' stress associated with various components of the application process. Remarkably, nearly half (48%) of applicants reported being extremely or very stressed when choosing the number of programs to which to apply. Sixteen and nineteen percent of applicants reported being extremely and very stressed, respectively, with regard to the overall cost. Eight and twenty‐two percent of applicants reported being extremely and very stressed, respectively, with regard to preparing application materials. Notably, no significant difference in mean (standard deviation [SD]) numerically converted stress levels due to overall cost was found by gender (2.9 [1.4] female vs 2.9 [1.3] male, *p* = .88), race (2.6 [1.1] white, 2.9 [1.6] Asian, 2.0 [1.4] black, 2.6 [1.4] Hispanic/American Indian and Alaska Native (AIAN)/mixed, *p* = .81), and access to home ENT program (2.5 [1.4] no home program vs 2.95 [1.3] home program, *p* = .44]. Similarly, no significant difference in mean (SD) numerically converted stress levels due to preparing application materials was found by gender (2.9 [1.1] female vs 2.9 [1.0] male, *p* = 0.92), race (2.6 [0.9] white, 3.0 [1.1] Asian, 2.5 [0.7] black, 3.3 [1.0] Hispanic/AIAN/mixed, *p* = .23), and access to home ENT program (2.7 [0.8] no home program vs 3.0 [1.1] home program, *p* = .51). Applicants reported relatively less stress with regards to choosing (25%) and ranking (22%) available rotation dates.

**Figure 4:**
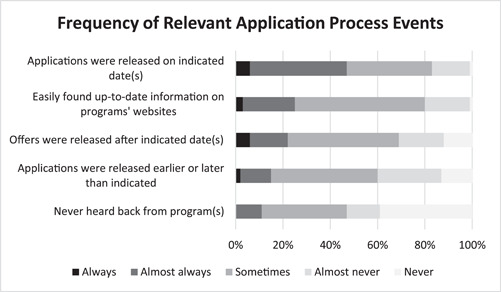
Graphical representation of the frequency of relevant away subinternship application process events as reported by survey respondents (n = 64).

Frequencies of various application process events are displayed in Figure 4. While nearly half (47%) of applicants reported that applications were almost always or always made available on indicated dates, there were more inconsistencies regarding offer releases. Nearly half (47%) reported that offers were sometimes released after the indicated date, and 11% of applicants almost never heard back from programs. Notably, 76% of applicants reported difficulty finding updated information on program websites at least some of the time.

**Figure 5:**
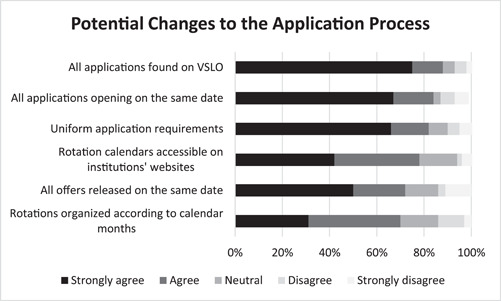
Graphical representation of levels of agreement to potential changes to the away subinternship application process as reported by survey respondents (n = 64). VSLO, Visiting Student Learning Opportunities.

Figure 5 shows applicants' opinions regarding various potential changes to the application process. The majority of applicants indicated agreement with every listed change, with the greatest support for making all applications available on VSLO (88%), uniform application release date (84%), and uniform application requirements (82%). Other proposed changes included a uniform offer release date (72%) and organizing every rotation according to calendar months (70%).

Free‐response comments from 21 applicants demonstrated similar themes regarding areas of frustration with the away subinternship application process. Themes included not knowing offer release dates (52%), fear of being “blacklisted” from programs after withdrawing applications and/or rejecting offers (29%), concerns about rotation logistics such as cost and housing (24%), outdated program websites (15%), variability in application requirements (14%), and variability in application release dates (14%).

## Discussion

OHNS is a competitive specialty. Away subinternships allow programs and applicants to assess “fit” and increase the likelihood of a successful match. In a recent study, 100% of surveyed 4th‐year medical students interested in OHNS from 10 US medical schools completed an away subinternship.^13^ Programs consistently report personal knowledge of applicants as among the most important criteria used when ranking applicants, and in cases of exceptional “fit,” match rates as high as nearly 50% are reported.^4^, ^6^, ^14^ Despite the universality of away subinternships, studies assessing the process of obtaining such experiences are lacking. To our knowledge, this study is the first analysis of available information and applicants' impressions regarding the OHNS away subinternship application process.

The AAMC's VSLO program was created, in part, to simplify the away subinternship application process. However, despite 80% of OHNS programs utilizing VSLO, many survey respondents commented on the difficulty of finding accurate information regarding elective opportunities, including offer release dates and program websites. A portion of students' dissatisfaction with the application process may be attributed to the 20% of OHNS programs that have yet to utilize VSLO, around half of which are military or osteopathic residency programs. While military programs utilize their own vetting process, which VSLO may not be suited for, it is unclear why osteopathic programs underutilize VSLO. Additionally, several allopathic residency programs do not utilize VSLO, including programs that traditionally receive abundant student interest, highlighting the need for streamlining access. Unsurprisingly, when asked about potential changes, having all applications for away subinternships available on VSLO was supported by most respondents (88%).

Among programs that utilize VSLO, varying application requirements, deadlines, and fees contribute to increased applicant stress. Respondents indicated most difficulty finding when applications open and when offers are released. Our analysis found widely varying application release dates and requirements, suggesting that application contents still differ tremendously among programs utilizing VSLO. Moreover, many applications on VSLO contain outdated information and schedules, including rotation calendars and rotation sites, requiring prospective applicants to search for more accurate information on program‐specific websites that are often also outdated. Unfortunately, inadequate program webpages are not unique to OHNS, suggesting that programs across all specialties must continually update webpages to ensure their accuracy and utility for prospective students.^10^, ^15^

Applicants also expressed frustration with the lack of transparency. Even when application and offer release dates were published, a substantial number of respondents reported applications opening and offers being released on dates different than initially indicated. Especially concerning is that 11% of applicants reported almost never hearing back from programs regarding their application status. Unsurprisingly, the most commonly reported concerns and stressors are regarding the number of applications to submit to guarantee enough offers in the setting of not knowing when offers would be released. Given the importance of away subinternships in the Match process and the competitiveness of the specialty, students often apply to many programs to optimize their chances of securing away subinternships. In our survey, applicants applied to up to 12 programs, despite wanting to do a maximum of 4 away subinternships. Because application submission status is often not known, students focus on securing any away subinternship rather than one(s) that are potentially a good “fit” (eg, preferred geographic location). If a less desired program notifies applicants first, applicants must either accept, foregoing a better “fit,” or decline, possibly alienating that program in the Match with no guarantee of acceptance from other, more desired away subinternships.^16^ Similarly, when a better “fit” program responds in the affirmative at a later date, applicants often struggle with the decision to decline previously accepted offers to secure the best‐fit subinternship.

The lack of transparency, uniformity, and availability of accurate away subinternship information makes planning difficult for prospective applicants. A significant number of surveyed respondents favored uniform dates for when applications open and for when offers are released in addition to uniform application requirements. A recent study highlighted the successful implementation of standardized deadlines within the OB‐GYN residency application process, which was well‐received by applicants and programs alike.^17^ A similar process of universal interview offer dates was adopted by the Otolaryngology Program Director's Association and was also well‐received by both programs and applicants. Standardizing away subinternship application and acceptance timelines via VSLO would likely be similarly beneficial.

Although it may be possible to standardize both when applications open and when offers are released, creating uniform application requirements poses a larger challenge given hospital‐specific requirements, such as drug screens and TB testing. While hospital and state policies for healthcare workers may be difficult to change, requirements like letters of recommendation and personal statements are program‐specific and lend themselves more nicely to standardization. Such requirements add significant time and effort to an already stressful process that occurs during a busy time in medical school. Many surveyed respondents suggested that these requirements may dissuade them from applying to particular electives. In as much as standardizing application requirements is feasible per specific medical school requirements, it should be a serious consideration toward facilitating the process for applicants.

Our study has several limitations. Despite efforts to broadly distribute our survey through Otomatch and program directors, our response rate is low at 13%. Furthermore, the true response rate is difficult to measure as complete applicant data for the 2023 Match cycle were not yet available, and we utilized ERAS 2023 preliminary data as an estimate. If the number of applicants during the current cycle was lower than the previous one, the percent response rate could actually be higher. Our survey was administered in May 2022, potentially limiting applicants' recall of the application process, which typically occurs in March and April. Additionally, we could not differentiate DO and MD respondents, precluding assessment of factors specifically affecting DO applicants. Another potential limitation related to the way the survey was disseminated is that the survey under‐represents applicants with no otolaryngology home program. As with all survey‐based studies, sample bias and response bias potentially limit the generalizability of our results. Programs may have had more information available regarding away subinternship application processes and requirements, including applications available on VSLO or separate websites, but we could not find them. Beyond VSLO and residency program websites, applicants could have gathered information about the application process from other sources, including mentorship from faculty and senior students and online forums, like Otomatch, all of which our study did not assess.

## Conclusion

The otolaryngology away subinternship application process is a considerable source of anxiety for medical student applicants, largely due to the tremendous variability in application and acceptance procedures. We recommend the following changes to improve the away subinternship application process: all applications on VSLO, uniform application requirements, and uniform application opening and offer release dates. While these changes require programs to adapt their current procedures, recent changes to residency application processes, including uniform interview offer dates and signaling, suggest that change is possible. The proposed recommendations would increase uniformity and transparency and could allow applicants to make more thoughtful decisions based on their priorities, ultimately benefiting both applicants and otolaryngology residency programs.

## Author Contributions

**Thomas Cyberski**, design, conduct, analysis, and presentation; **Swetha Tatineni**, design, conduct, analysis, and presentation; **Andrea Shogan**, design, analysis, and presentation; **Fuad M. Baroody**, design, analysis, and presentation.

## Disclosures

### Competing interests

None.

### Funding source

None.

## Supporting information

Survey questions assessing students' perceptions of the away sub‐internship application process.

## References

[1] NRMP . NRMP releases the 2022 Main Residency Match Results and Data publication, the most comprehensive data resource for the Main Residency Match®. June 1, 2022. Accessed December 21, 2022. https://www.nrmp.org/about/news/2022/06/nrmp-releases-the-2022-main-residency-match-results-and-data-publication-the-most-comprehensive-data-resource-for-the-main-residency-match/

[2] Girard AO , Qiu C , Lake IV , Chen J , Lopez CD , Yang R . US medical student perspectives on the Impact of a pass/fail USMLE step 1. J Surg Educ. 2022;79(2):397‐408. 10.1016/j.jsurg.2021.09.010 34602379

[3] Pletcher SD , David Chang CW , Thorne MC , Malekzadeh S . The otolaryngology residency program preference signaling experience. Acad Med. 2022;97:664‐668. 34618735 10.1097/ACM.0000000000004441 PMC9028299

[4] Thomas CM , Cabrera‐Muffly C . Otolaryngology externships and the match: productive or futile? Laryngoscope. 2017;127(10):2242‐2246. 10.1002/lary.26639 28714544

[5] Villwock JA , Hamill CS , Ryan JT , Nicholas BD . The role of the away rotation in otolaryngology residency. Otolaryngol Head Neck Surg. 2017;156(6):1104‐1107. 10.1177/0194599817698431 28349746

[6] National Resident Matching Program . National Resident Matching Program, Data Release and Research Committee: Results of the NRMP Program Director Survey. National Resident Matching Program; 2021.

[7] Blood T , Hill K , Brown S , Mulcahey MK , Eberson CP . Variability of the orthopaedic away rotation: a survey of orthopaedic program directors. J Am Acad Orthop Surg Glob Res Rev. 2021;5(3):e21.00024. 10.5435/JAAOSGlobal-D-21-00024 PMC7954370 33720055

[8] Zhang XC , Gordon D , Lutfy‐Clayton L , et al. Away rotation applications in emergency medicine: medical student behaviors, outcomes, and stressors. J Emerg Med. 2022;62(3):401‐412. 10.1016/j.jemermed.2021.11.008 35078704

[9] Drolet BC , Brower JP , Lifchez SD , Janis JE , Liu PY . Away rotations and matching in integrated plastic surgery residency: applicant and program director perspectives. Plast Reconstr Surg. 2016;137(4):1337‐1343. 10.1097/PRS.0000000000002029 27018690

[10] Rai R , Sabharwal S . Availability and quality of online information on sub‐internships in U.S. orthopaedic residency programs. JBJS Open Access. 2019;4(1):e0036. 10.2106/JBJS.OA.18.00036 31161150 PMC6510465

[11] Green EH , Hershman W , Sarfaty S . The value of the subinternship: a survey of fourth year medical students. Med Educ Online. 2004;9(1):4349. 10.3402/meo.v9i.4349 28253132

[12] AAMC . FACTS: applicants and matriculants data. 2022. Accessed December 21, 2022. https://www.aamc.org/data-reports/students-residents/interactive-data/2022-facts-applicants-and-matriculants-data

[13] Higgins E , Newman L , Halligan K , Miller M , Schwab S , Kosowicz L . Do audition electives impact match success? Med Educ Online. 2016;21:31325. 10.3402/meo.v21.31325 27301380 PMC4908064

[14] Lenze NR , Mihalic AP , DeMason CE , et al. Predictors of otolaryngology applicant success using the Texas STAR database. Laryngoscope Investig Otolaryngol. 2021;6(2):188‐194. 10.1002/lio2.549 PMC8035942 33869750

[15] Skovrlj B , Silvestre J , Ibeh C , Abbatematteo JM , Mocco J . Neurosurgery residency websites: a critical evaluation. World Neurosurgery. 2015;84(3):727‐733. 10.1016/j.wneu.2015.04.051 25940210

[16] Griffith M , DeMasi SC , McGrath AJ , Love JN , Moll J , Santen SA . Time to reevaluate the away rotation: improving return on investment for students and schools. Acad Med. 2019;94:496‐500. 30379660 10.1097/ACM.0000000000002505

[17] Hammoud MM , Winkel AF , Strand EA , et al. Stakeholder perspectives on standardizing the residency application and interview processes. J Surg Educ. 2021;78(4):1103‐1110. 10.1016/j.jsurg.2020.11.002 33199253

