# Supplementary material for: Otolaryngology Away Subinternships: An Analysis of the Application Process and Survey of Current Applicants' Perspectives
Source: OTO Open. 2023 Mar 28;7(1):e49. doi: 10.1002/oto2.49 (PMC10046704; doi:10.1002/oto2.49)
Supplement: Supplementary file 1 — Survey questions assessing students' perceptions of the away sub‐internship application process. [file OTO2-7-e49-s001.pdf]

Otolaryngology Away Subinternships Application Process Survey

Thank you for participating in our survey. This survey should take approximately 10 minutes to complete. Results from the survey will be reported only in aggregate form and will NOT be associated with you or your medical school. This survey aims to assess your experience of applying to in-person otolaryngology away subinternships for the 2022 - 2023 academic year. Please note, you must be a MS4 during the 2022 - 2023 academic year and applying for IN-PERSON away subinternship(s) to be eligible to complete the survey.

This survey was created by rising MS4s at the University of Chicago to evaluate possible challenges while applying to otolaryngology away subinternships. We are hoping to gauge any difficulties that our peers might experience during this process. We hope to shed more light on the application process with the aim of advocating for productive changes to improve the experiences of future Otolaryngology applicants.

If you have questions about this study, please contact Fuad Baroody at fbaroody@surgery.bsd.uchicago.edu. This study has been approved by the University of Chicago IRB.

What year are you in medical school during the 2022 - 2023 academic year? (only rising MS4s are eligible)	◯ M1<br>◯ M2<br>◯ M3<br>◯ M4<br>◯ Year-off (research, master's program, etc)<br>◯ PhD portion of the MD/PhD program
To which gender identity do you most identify?	◯ Female<br>◯ Male<br>◯ Non-binary<br>◯ Self-describe<br>◯ Prefer not to say
How do you self describe your gender identity?	__________________________________
Are you of Hispanic, Latino, or Spanish origin?	◯ Yes<br>◯ No
How would you describe yourself? (Check all that apply)	☐ American Indian or Alaska Native<br>☐ Asian<br>☐ Black or African American<br>☐ Native Hawaiian or Pacific Islander<br>☐ White<br>☐ Other<br>☐ Prefer not to say
Please describe "other"	__________________________________
What is your age?	__________________________________
What is your marital status?	◯ Single, never married<br>◯ Married or domestic partnership<br>◯ Widowed<br>◯ Divorced<br>◯ Separated<br>◯ Prefer not to say

How many children do you have?	◯ 0<br>◯ 1<br>◯ 2<br>◯ 3+
Region of home medical school	◯ Central (IL, IN, IA, KS, MI, MN, MO, NE, ND, OH, SD, WI)<br>◯ Northeastern (CT, DE, ME, MD, MA, NH, NJ, NY, PA, RI, VT, DC)<br>◯ Western (AK, AZ, CA, CO, HI, ID, MT, NV, NM, OR, UT, WA, WY)<br>◯ Southern (AL, AR, FL, GA, KY, LA, MS, NC, OK, PR, SC, TN, TX, VA, WV)
Does your home medical school have an ENT program?	◯ Yes<br>◯ No
How many away rotations do you plan on doing?	______________________
How many institutions did you apply to?	______________________
First choice region to do an away subinternship	◯ Central (IL, IN, IA, KS, MI, MN, MO, NE, ND, OH, SD, WI) ◯ Northeastern (CT, DE, ME, MD, MA, NH, NJ, NY, PA, RI, VT, DC) ◯ Western (AK, AZ, CA, CO, HI, ID, MT, NV, NM, OR, UT, WA, WY) ◯ Southern (AL, AR, FL, GA, KY, LA, MS, NC, OK, PR, SC, TN, TX, VA, WV)

Which factors did you consider in choosing which away subinternship(s) to apply to? (choose and RANK top 5 in descending order of importance)

	1	2	3	4	5
Region(s) where you would like to match	◯	◯	◯	◯	◯
Prestige of programs	◯	◯	◯	◯	◯
Size of programs	◯	◯	◯	◯	◯
Diversity of residents/faculty within the program	◯	◯	◯	◯	◯
Research track	◯	◯	◯	◯	◯
Available rotation dates	◯	◯	◯	◯	◯
Proximity to home medical school	◯	◯	◯	◯	◯
Having friends/family in the area(s)	◯	◯	◯	◯	◯
Familial obligations/children	◯	◯	◯	◯	◯
Cost of living in the area(s)	◯	◯	◯	◯	◯
Cost of application	◯	◯	◯	◯	◯
Previous personal connection to the institution(s)	◯	◯	◯	◯	◯
Suggested/facilitated by home program leadership	◯	◯	◯	◯	◯
Prior exposure to program(s) (social media, internet, information sessions, etc)	◯	◯	◯	◯	◯
Other (please specify)	◯	◯	◯	◯	◯

Please specify

____

Please indicate your ability to find the following while applying for away subinternship(s).

	Very easy	Easy	Neutral	Difficult	Very difficult
Date on which applications open	◯	◯	◯	◯	◯
Date on or after which you can expect to receive offers	◯	◯	◯	◯	◯
Institutions' rotation calendars	◯	◯	◯	◯	◯
Primary sites of rotations	◯	◯	◯	◯	◯
Institutions' updated application for the 2022 - 2023 academic year	◯	◯	◯	◯	◯
Application requirements	◯	◯	◯	◯	◯
Contact persons	◯	◯	◯	◯	◯
Programs' websites	◯	◯	◯	◯	◯

Please indicate how stressed you felt (or would feel) with regards to the following while applying for away subinternship(s).

	Not at all stressed	Somewhat stressed	Moderately stressed	Very stressed	Extremely stressed
Choosing how many programs to apply to	○	○	○	○	○
Choosing the rotation dates to apply to for each program	○	○	○	○	○
Ranking the dates applied to for each program	○	○	○	○	○
Preparing application materials	○	○	○	○	○
Overall cost	○	○	○	○	○

What were your concerns while applying for away subinternships? (choose and RANK top 5 in descending order of importance)

	1	2	3	4	5
Applying to too few programs	◯	◯	◯	◯	◯
Applying to too many programs	◯	◯	◯	◯	◯
Not receiving enough offers	◯	◯	◯	◯	◯
Not knowing when applications would open	◯	◯	◯	◯	◯
Not knowing when offers would be released	◯	◯	◯	◯	◯
Withdrawing application before receiving an offer	◯	◯	◯	◯	◯
Declining an offer before acceptance	◯	◯	◯	◯	◯
Withdrawing after accepting offer	◯	◯	◯	◯	◯
Overall cost	◯	◯	◯	◯	◯
Other (please specify)	◯	◯	◯	◯	◯

Please specify. __________________________________

How often did the following occur?

	Never	Almost never	Sometimes	Almost always	Always
Easily found up-to-date information on programs' websites	○	○	○	○	○
Applications were released on indicated date(s)	○	○	○	○	○
Applications were released earlier or later than indicated	○	○	○	○	○
Offers were released after indicated date(s)	○	○	○	○	○
Never heard back from program(s)	○	○	○	○	○

I believe the following would have improved the away subinternship application process.

	Strongly agree	Agree	Neutral	Disagree	Strongly disagree
Rotations organized according to calendar months	○	○	○	○	○
Rotation calendars accessible on institutions' websites	○	○	○	○	○
All applications opening on the same date	○	○	○	○	○
All applications found on VSLO	○	○	○	○	○
Uniform application requirements	○	○	○	○	○
All offers released on the same date	○	○	○	○	○

Do you have any additional thoughts you would like to share?
